# Ventilator-Associated Pneumonia and Its Responsible Germs; an Epidemiological Study

**Published:** 2017-01-10

**Authors:** Rama Bozorgmehr, Vanousheh Bahrani, Alireza Fatemi

**Affiliations:** 1 Clinical Research Development Unit, Shohadaye Tajrish Hospital, Shahid Beheshti University of Medical Sciences, Tehran, Iran.; 2 Infectious Diseases and Tropical Medicine Research Center, Shahid Beheshti University of Medical Sciences, Tehran, Iran.

**Keywords:** Pneumonia, ventilator-associated, cross infection, drug resistance, microbial, intensive care units

## Abstract

**Introduction:**

Ventilator-associated pneumonia (VAP) is one of the most common hospital infections and a side effect of lengthy stay in intensive care unit (ICU). Considering the ever-changing pattern of common pathogens in infectious diseases and the raise in prevalence of hospital infections, the present study was designed aiming to determine the prevalence of VAP and its bacterial causes.

**Methods:**

In this cross-sectional study, the medical profiles of all the patients under mechanical ventilation, who had no symptoms of pneumonia at the time of intubation and developed new infiltration in chest radiography after 48 hours under mechanical ventilation along with at least 2 of the symptoms including fever, hypothermia, leukocytosis, leukopenia, or purulent discharge from the lungs, were evaluated. Demographic data, clinical and laboratory findings, and final outcome of the patients were extracted from the patient’s clinical profile and reported using SPSS version 20 and descriptive statistics.

**Results:**

518 patients with the mean age of 62.3 ± 20.8 years were evaluated (50.9% female). Mean time interval between intubation and showing symptoms was 10.89 ± 12.27 days. Purulent discharges (100%), leukocytosis (71.9%), fever (49.1%), hypothermia (12.3%), and leukopenia (8.8%) were the most common clinical and laboratory symptoms and acinetobacter baumannii (31.58%) and klebsiella pneumoniae (29.82%) were the most common germs growing in sputum cultures. 19 (33.3%) cases of pan drug resistance (PDR) and 10 (17.5%) cases of extensive drug resistance (XDR) were seen. Mortality due to VAP was 78.9% and there was no significant correlation between age (p = 0.841), sex (p = 0.473), ICU admission (p = 0.777), duration of hospitalization (p = 0.254), leukocytosis (p = 0.790), leukopenia (p = 0.952), fever (p = 0.171), hypothermia (p = 0.639), type of culture (p = 0.282), and type of antibiotic resistance (p = 0.066) with mortality.

**Conclusion:**

Prevalence of VAP and its associated mortality were 11% and 78.9%, respectively. The most common symptoms and signs were purulent discharge, leukocytosis, and fever. Acinetobacter baumannii and klebsiella pneumoniae were the most common germs in sputum cultures with 50% resistance to commonly used antibiotics.

## Introduction

The respiratory infection caused by micro-aspiration of organisms, 48 hours after going under mechanical ventilation is called ventilator-associated pneumonia (VAP) (1). Pneumonia is one of the most common hospital infections and a side effect of long stay in the intensive care unit (ICU) (2). This problem has a prevalence of 16 to 78%, while infections of urinary tract or skin have a prevalence of 1 – 4% (2-6). Due to the important role of antibiotic-resistant bacteria in these kinds of infection, with longer duration of hospital stay, the probability of mortality will rise (3, 7, 8). VAPis the reason for increased length of stay in ICU and is responsible for about 50% of antibiotic prescriptions in this unit. The risk of this infection has been reported to be 3% for each day of intubation during the first 5 days, 2% for each day during 5^th^to 10^th^ day, and 1% after 10 days of intubation (9). Among the most common germs responsible for VAP are staphylococcus aureus, pseudomonas aeruginosa, klebsiella pneumoniae, and acinetobacter (4, 10). In a study on 107 patients under mechanical ventilation, prevalence of VAP was calculated to be 28.54%, with pseudomonas aeruginosa, meticillin resistant staphylococcus aureus, klebsiella pneumoniae, and acinetobacter baumannii (6). In another study, the total rate of hospital pneumonia was found to be 26.2% and its mortality rate was estimated to be 78.8% (11). A study in Shiraz, Iran, also reported a 10.2% VAP rate and acinetobacter baumannii as the most common responsible organism (12). 2 other studies reported prevalence of VAP to be 21.6% and 16% in 2013 and 2014, respectively (13, 14). Considering the changes in pattern of common pathogens in infectious diseases and raise in hospital infection rates shown in various studies, identifying the pathogens responsible for infections can be of great help in selecting the proper treatment for VAP and is therefore a requirement and research priority all over the world. The importance of this issue is emphasized when we note that most of these patients have been intubated in emergency department and stayed there for a while. Therefore, the present study aimed to determine the rate of VAP and its bacterial causes.

## Methods

***Study design and setting***

The present study is a retrospective cross-sectional one carried out on patients under mechanical ventilation in ICU or other departments of Shohadaye Tajrish Hospital, Tehran, Iran, during 2014-2016, aiming to determine the prevalence of VAP and its bacterial causes in these patients. Protocol of this study was approved by the ethics committee of Shahid Beheshti University of Medical Sciences. The researchers adhered to confidentiality of patient data and Declaration of Helsinki principles.

***Participants***

All the patients that had no signs of pneumonia at the time of intubation who developed new infiltration in chest radiography after 48 hours of intubation along with at least 2 of the symptoms including fever, hypothermia, leukocytosis, leukopenia, or purulent discharge from the lungs were considered VAP cases and included in the study. If the patients had pneumonia before going under ventilation or during the initial 48 hours or their clinical data were not available, they were excluded. No age or sex limitation was applied and the reason for intubation was not considered in exclusion criteria. Consecutive sampling was used.

***Data gathering***

Demographic data of the patients (age, sex, admission ward), results of blood cell count, smear and culture of respiratory secretions, type of microorganism that grow, antibiotic resistance, clinical symptoms, chest radiography findings, computed tomography (CT) scan of lungs, if present, and final outcome of the patients were extracted from their medical profile and recorded in a checklist designed for this purpose. Data were gathered by a senior resident of internal medicine and when in doubt, an infectious disease specialist or an internal medicine specialist was consulted. The findings of patients’ radiography were reported by the center’s radiologist or the pulmonologist in charge of patient management in ICU. 

***Statistical analysis***

Required sample size for this study was calculated to be 456 cases considering the 5% probability of VAP (3% for each day in the initial 5 days and 2% for each day 5-10 days after intubation), α = 5% and desired precision of 2% (15). Finally, data were analyzed using SPSS version 20 and Chi square and t-test. Qualitative data were reported as percentage and frequency, and quantitative ones as mean ± standard deviation (SD). To evaluate the correlation between studied variables and final outcome (mortality) chi-square test was used. P < 0.05 was considered as significance level.

## Results

***Baseline characteristics***

518 patients with the mean age of 62.3 ± 20.8 (range: 15 – 97) years underwent mechanical ventilation via orotracheal intubation during the study period (50.9% female). 57 (11%) of cases developed VAP. Demographic data, clinical symptoms, and laboratory findings of the studied patients are summarized in table 1. The highest frequency of patients belonged to ≥ 75 years age group (30.19%). Mean time interval between intubation and showing symptoms was 10.89 ± 12.27 days (range: 2 – 80). Only 21 (36.8%) patients were hospitalized in ICU and others were admitted to other departments. Purulent discharges with 100%, leukocytosis with 71.9%, fever with 49.1%, hypothermia with 12.3%, and leukopenia with 8.8% were the most common clinical and laboratory findings in the studied patients.

***Cultures***

Tables 2 and 3 depict the most common germ growths in sputum culture and the results of antibiogram done for patients with VAP. The most common germ growths belonged to acinetobacter baumannii with 31.58%, and klebsiella pneumoniae with 29.82%, respectively. Rate of resistance to ciprofloxacin, doxycycline, cotrimoxazole, ceftazidime, and cefotaxime were reported to be more than 50%. Finally, 19 (33.3%) cases of pan drug resistance (PDR) and 10 (17.5%) cases of extensive drug resistance (XDR) were reported. No significant correlation was detected between the type of germ growth in sputum culture and presence of PDR or XDR (p = 0.931). Radiography of 100% of the patients included turbidity in 1 or multiple lobes. 

***Outcome***

Mortality due to VAP in the present study was estimated to be 78.9% (45 cases). No significant correlation was seen between age (p = 0.841), sex (p = 0.473), ICU admission (p = 0.777), duration of hospitalization (p = 0.254), leukocytosis (p = 0.790), leukopenia (p = 0.952), fever (p = 0.171), hypothermia (p = 0.639), type of culture (p = 0.282), and type of antibiotic resistance (p = 0.066) with mortality.

**Table 1:** Demographic and clinical data of studied patients

**Variables **	**Number (%)**
**Age (year)**	
15 - 29.9	6 (11.32)
30 – 44.9	4 (7.55)
45 – 59.9	12 (22.64)
60 -74.9	15 (28.30)
>75	16 (30.19)
Sex	
Female	29 (50.88)
Male	28 (49.12)
**Hospitalized in**	
Intensive care unit	21 (36.64)
Other departments	36 (63.16)
**Fever **	
Yes	28 (49.12)
No	29 (50.88)
**Hypothermia **	
Yes	7 (12.28)
No	50 (87.72)
**Purulent discharge**	
Yes	57 (100)
No	0 (0)
**Leukocytosis **	
Yes	41 (71.93)
No	16 (28.07)
**Leukopenia **	
Yes	5 (8.77)
No	52 (91.23)
**Drug resistance**	
Pan drug resistance (PDR)	19 (65.52)
Extensive drug resistance (XDR)	10 (34.48)
**Final outcome**	
Recovery	12 (21.05)
Death	45 (78.95)

**Table 2:** Frequency of germs’ growth in studied patients’ sputum culture

**Growing germ**	**Number (%)**
**Acinetobacter Baumannii**	18 (31.6)
**Klebsiella Pneumoniae**	17 (29.8)
**Enterobacteriaceae**	7 (12.3)
**Candida Albicans**	4 (7)
**Pseudomonas Aeruginosa**	4 (7)
**Staphylococcus Aureus**	3 (5.3)
**Escherichia Coli**	2 (3.5)
**Proteus Mirabilis**	1 (1.8)
**Negative**	1 (1.8)

**Table 3:** Frequency of antibiotic resistance in studied patients

**Type of antibiotic**	**Number (%)**
**Ciprofloxacin**	42 (73.7)
**Doxycycline **	36 (63.2)
**Cotrimoxazole**	32 (56.1)
**Ceftazidime**	31 (54.4)
**Cefotaxime**	31 (54.4)
**Meropenem**	28 (49.1)
**Imipenem**	28 (49.1)
**Amikacin**	26 (45.6)
**Clindamycin **	22 (38.6)
**Tobramycin **	21 (35.1)
**Cefepime**	20 (31.8)
**Colistin**	18 (31.6)
**Ampicillin sulbactam**	13 (22.8)
**Levofloxacin **	9 (15.8)
**Cephalexin **	6 (10.5)
**Piperacillin**	6 (10.5)
**Gentamicin **	5 (8.8)
**Ceftriaxone **	4 (7)
**Cefixime**	3 (5.3)
**Azithromycin **	2 (3.5)
Ofloxacin	1 (1.8)

## Discussion

Based on our findings, prevalence of VAP was 11% among the studied patients and mortality due to it was estimated to be 78.9%. The most common clinical and laboratory symptoms and signs of this type of pneumonia were purulent discharge, leukocytosis, and fever. Acinetobacter baumannii and klebsiella pneumonia were the most common germs growing in sputum cultures that showed resistance to commonly used antibiotics in more than 50% of cases. In a study by Nadi et al. mean age of the population affected with pneumonia in 353 patients who were hospitalized in ICU of Be’sat and Ekbatan Hospitals, Hamedan, Iran, was 51.2 ± 21.9 years, only 36 (10.2%) of which were affected with VAP (16). As mentioned before, health care providers should face a big challenge named hospital infections. In the United States, every year, an average of about 2 million people are affected with this kind of infection and 90000 die, which makes hospital infections the 5^th ^most common cause of death in health centers (15). The most common hospital infections are reported to be respiratory (65%), urinary tract (17%), and blood (12%) infections (15). Ventilator associated infections have the highest rate of mortality among hospital acquired infections, 90% of which occur during mechanical ventilation and 50% in the initial 4 days of going under mechanical ventilation (15). 

In 2009, it was estimated that VAP increases days in need of ventilator by 9.6 days, length of stay in ICU by 6.1 days, and hospitalization duration by 11.5 days, which inflicts physical and financial burdens on the patient and health care system (17). Despite measures taken and recent advances in treatment, prevention, and management of pathogens associated with ventilator, pneumonia is still a major cause of death in hospitalized patients. Since multi-drug resistant pathogens play a major role in VAP, currently antibiotics that properly cover these pathogens are used. These antibiotics include 3^rd^ or 4^th^ generation of cephalosporin (ceftazidime, cefepime), β-lactamase inhibitors (piperacillin, tazobactam), carbapenems (imipenem, meropenem) in combination with an aminoglycoside (gentamicin, tobramycin, amikacin) or an anti-peseudomonas fluoroquinolone (levofloxacin, ciprofloxacine) (15). In the present study, VAP had a prevalence of 11% among patients under mechanical ventilation. This rate was reported to be 19% in ICU of Shahid Beheshti Hospital, Kashan, Iran, during 2009-2010. In that study, a significant correlation was found between age, Glasgow coma scale, positioning patient’s head in 30 or more degrees, oral hygiene, and training the staff regarding infection control with hospital pneumonia; and continuous training of staff and taking complete and regular care of oral hygiene of patients under mechanical ventilation were recommended (18).

In a study by Chung et al., 77.3% of intubated patients admitted to ICU had VAP (19). In a similar study, Klompas et al. reported the prevalence of VAP to be 9.5% among 599 patients who were hospitalized in ICU of a hospital in America (20). This difference in prevalence may be due to the method of care given, the skill and experience of nurses, adhering to standards and beds being physically standard, positioning of patient’s head, space between beds and their rate of occupation, use of gloves and gowns by staff, oral hygiene, prevention of stomach distension, and method of prophylactic antibiotic use. Therefore, the higher the rate of standard treatments carried out for intubated patients, the lower the rate of pneumonia. In the present study, there was no significant difference between ICU of departments (internal medicine and surgery) regarding incidence of pneumonia or final outcome of mortality. In a study in Arak carried out in 2011, there was a significant difference between ICU of internal medicine and surgery wards (21). In addition, in the Klompas et al. study, prevalence of pneumonia was 33% in internal medicine ward and 61% in surgery wad. This difference might be due to more invasive procedures, catheterization, intubation duration, and higher length of stay in surgery ward compared to internal medicine ward (20). The most common pathogens extracted from patients in the present study were acinetobacter baumannii and klebsiella pneumoniae, while in another study the most common pathogens were acinetobacter, staphylococcus aureus, and pseudomonas aeruginosa (21). In 2 separate studies, common germs responsible for pneumonia were staphylococcus aureus, pseudomonas aeruginosa, klebsiella pneumoniae, andacinetobacter, which is somehow similar to the present study (4, 10). In addition, in pediatric ICU, the most common germs in sputum of pneumonia patients were staphylococcus aureus, pseudomonas aeruginosa, and enterobacter (22). Regarding resistance to antibiotics in this study, pathogens were resistant to ciprofloxacin, doxycycline, cotrimoxazole, ceftazidime, and cefotaxime more than 50%. In a similar study, acinetobacter had the most resistance to gentamicin and was most sensitive to imipenem (21). Sadly, 78.9% of our studied patients died because of VAP. In a study by Zarinfar, 11.7% of the patient fully recovered, 18.3% were relatively better, 20% did not get better, and 50% died, which is lower than the rate in our study. As mentioned before, this might be due to the procedures done, method of intubation, physical health, positioning of patient’s head, and type of antibiotic prescribed (21).

***Limitation***

Among the limitations of this study were its retrospective design and extracting data from patients’ medical profile, which increase the probability of losing some information due to careless profile recording. In addition, data regarding the underlying disease and cause of intubation were not considered, while they may play a role in increasing mortality due to ventilation.

## Conclusion:

Based on our findings, prevalence of VAP and its associated mortality were 11% and 78.9%, respectively. The most common symptoms and signs were purulent discharge, leukocytosis, and fever. Acinetobacter baumannii and klebsiella pneumoniae were the most common germs in sputum cultures with 50% resistance to commonly used antibiotics.
